# Successful treatment with teduglutide in short bowel syndrome with intestinal failure secondary to small-bowel volvulus: a case report and literature review

**DOI:** 10.1097/RC9.0000000000000863

**Published:** 2026-08-17

**Authors:** Shingo Ito, Shota Akabane, Kei Hosoda, Shoichi Fujii

**Affiliations:** Department of Surgery, Shonan Kamakura General Hospital, Kamakura, Kanagawa, Japan

## Abstract

**Introduction and importance:**

Short bowel syndrome (SBS) is a major cause of intestinal failure (IF). Patients with SBS-associated IF (SBS-IF) often require long-term parenteral nutrition (PN) and suffer from complications that impair quality of life (QoL), including severe diarrhea, weight loss, sleep disturbances, and catheter-related bloodstream infections (CRBSIs). Teduglutide, a glucagon-like peptide-2 analog, enhances intestinal adaptation and reduces dependence on PN. However, evidence in patients with ultra-short residual bowel remains limited, particularly in Japan. We report a case of severe SBS-IF in which teduglutide enabled complete weaning from PN and substantial QoL improvement.

**Case presentation and clinical discussion:**

A 70-year-old man underwent an emergency, extensive small-bowel resection for small-bowel volvulus, leaving approximately 20 cm of small intestine with the colon in continuity. He developed SBS-IF requiring home PN and experienced watery diarrhea (more than 10 times per day), weight loss, sleep disturbance, and recurrent CRBSI. Four years after surgery, teduglutide was initiated. Within 4 months, stool consistency improved, bowel frequency decreased to 3–5 times per day, and absorption improved, allowing complete discontinuation of PN. Body weight stabilized, CRBSI resolved, and disease-specific QoL improved markedly.

**Conclusion:**

Even in ultra-short-bowel SBS-IF, teduglutide may achieve PN independence and clinically meaningful improvements in gastrointestinal symptoms, weight maintenance, and QoL.

## Introduction

Short bowel syndrome (SBS) is a rare but severe malabsorptive disorder resulting from extensive resection of the small intestine, congenital abnormalities, or disease-related loss of intestinal absorptive capacity. It represents one of the most common causes of intestinal failure (IF), which is defined as a reduction of gut function below the minimum required for the absorption of macronutrients, water, and electrolytes, necessitating intravenous supplementation to maintain health and survival^[^1^]^. Patients with SBS-associated IF (SBS-IF) often depend on long-term parenteral nutrition (PN), which is associated with substantial morbidity, including catheter-related bloodstream infections (CRBSI), liver dysfunction, metabolic complications, and a marked reduction in quality of life (QoL)^[^1–3^]^.

HIGHLIGHTS

- A patient with short bowel syndrome with intestinal failure (SBS-IF) has impaired quality of life (QoL) and suffers from severe complications.
- Teduglutide is a glucagon-like peptide-2 analog used in the treatment of SBS-IF.
- Teduglutide reduces parenteral nutrition dependence in patients with SBS-IF and improves SBS-QoL.

The clinical severity of SBS-IF is affected by multiple factors, including the length and anatomical segment of the remnant bowel, the presence of colon in continuity, and the adaptive capacity of the residual intestine. In adults, the most common etiologies of SBS-IF include Crohn’s disease, mesenteric ischemia, volvulus, radiation enteritis, and postoperative complications. Despite advances in nutritional support and intestinal rehabilitation, numerous patients with severe SBS remain permanently dependent on PN, and therapeutic options have historically been limited to symptomatic management aimed at controlling diarrhea and maintaining fluid and electrolyte balance^[^4^]^.

Glucagon-like peptide-2 (GLP-2) is an intestinotrophic hormone secreted by enteroendocrine L cells, predominantly located in the distal ileum and colon, in response to nutrient ingestion. GLP-2 improves intestinal adaptation by promoting mucosal growth, improving barrier function, increasing intestinal blood flow, and reducing gastric motility and secretion. Teduglutide is a recombinant GLP-2 analog resistant to enzymatic degradation and has been demonstrated to improve intestinal absorption and reduce PN requirements in patients with SBS-IF. Pivotal clinical trials, including the STEPS and STEPS-2 studies, have demonstrated that teduglutide significantly reduces PN volume and frequency, with some patients achieving partial or complete independence from PN^[^5–8^]^.

In Japan, teduglutide was approved for reimbursement for clinical use in 2019; however, there is limited real-world evidence concerning its effectiveness, particularly in patients with an extremely short residual bowel length and severe long-term complications. Moreover, although previous studies have focused primarily on reductions in PN volume, there is a scarcity of data assessing patient-reported outcomes, including disease-specific QoL. Herein, we report a case of severe SBS-IF secondary to small-bowel volvulus in which teduglutide treatment resulted in complete weaning off PN and marked improvement in SBS-specific QoL, thus emphasizing the potential clinical and patient-centered benefits of this therapy.

This manuscript was written according to the SCARE criteria^[^9^]^.

## Case presentation

**Figure 1.**
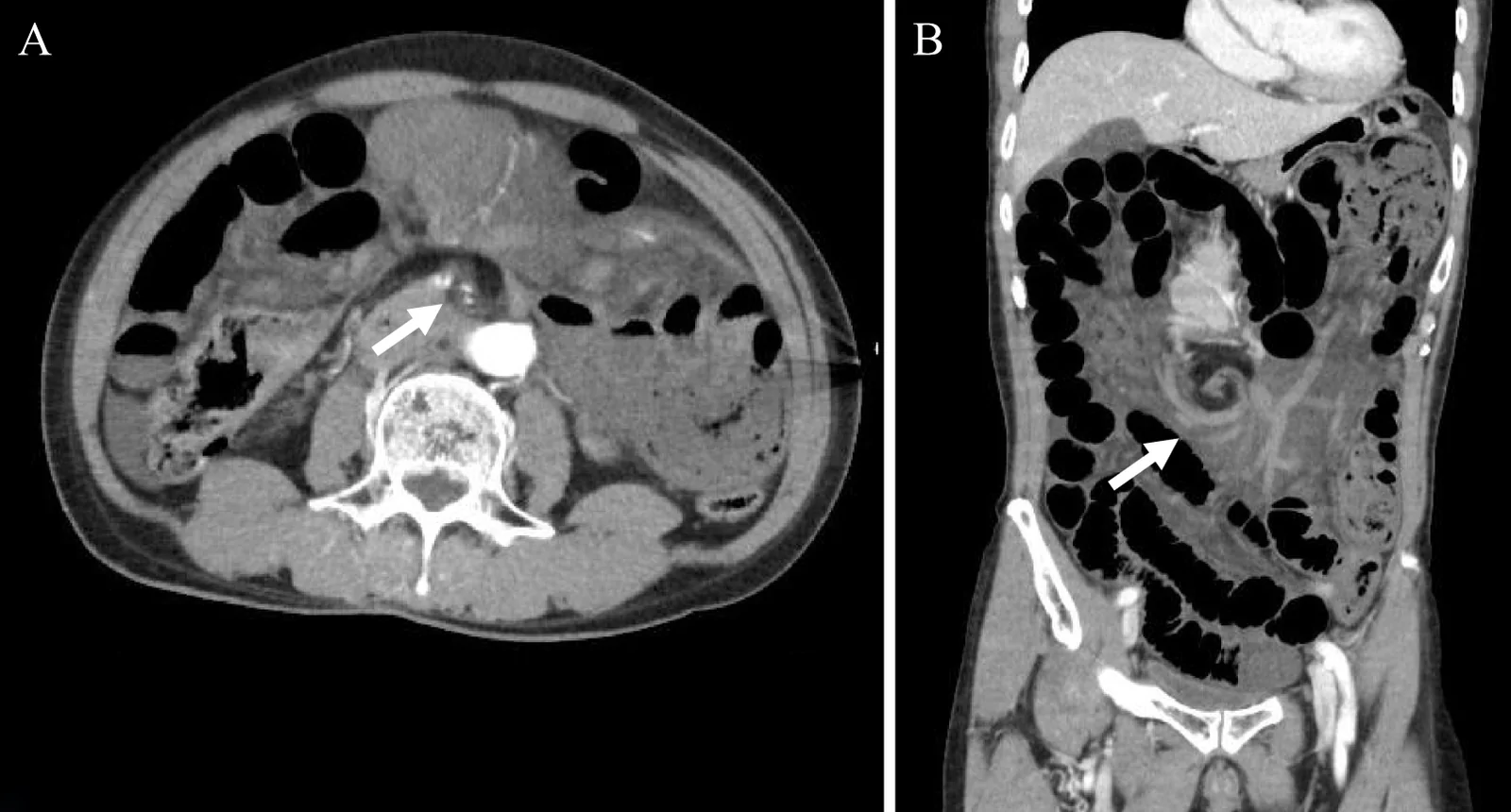
Contrast-enhanced computed tomography. (A) Axial plane; (B) coronal plane. The white arrows indicate the whirl sign in the mesentery, consistent with small bowel volvulus.

**Figure 2.**
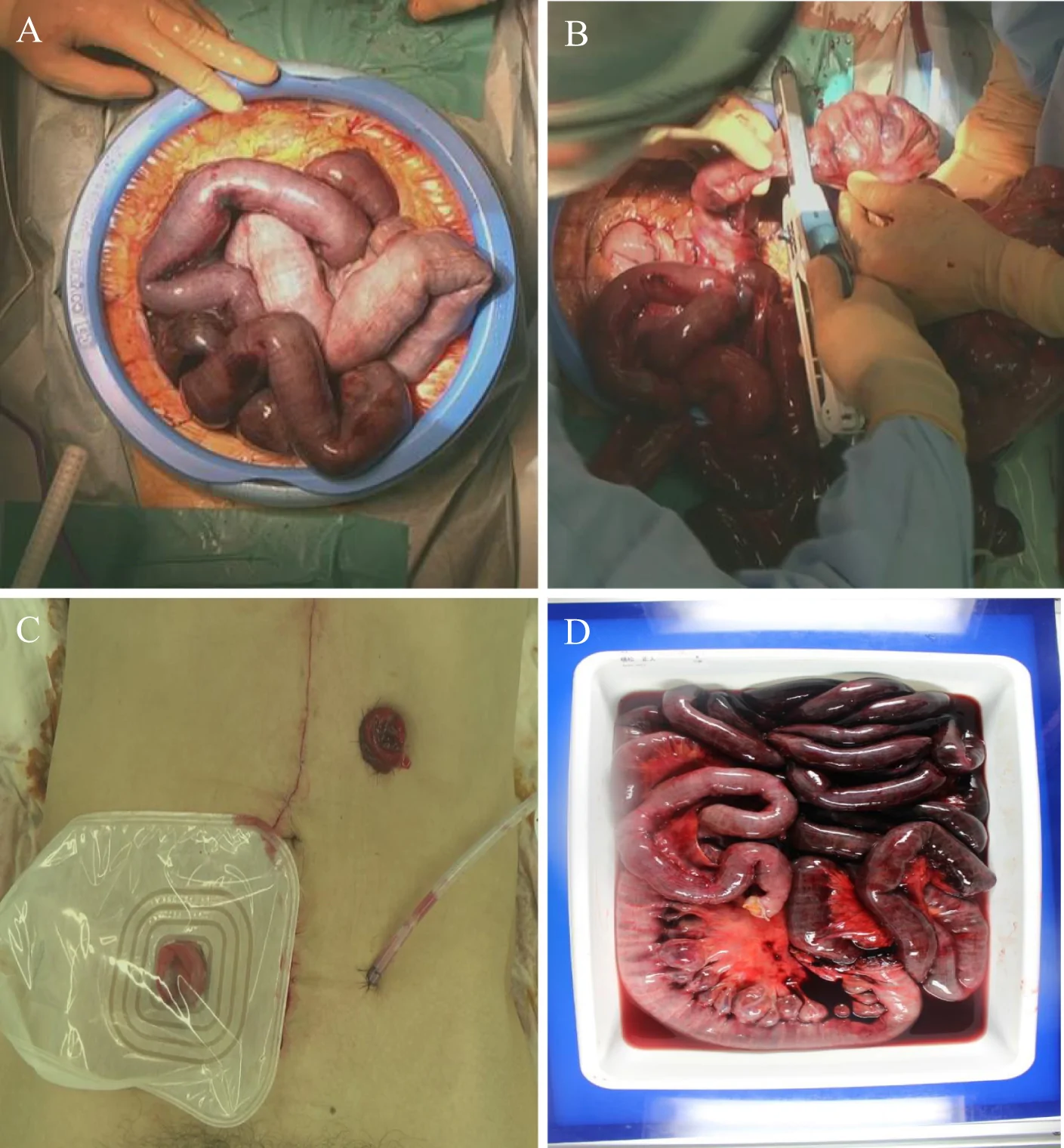
Intra-operative findings. (A) Twisted small bowel around the mesentery. (B) Resection of the extensive small intestine. (C) Jejunostomy and ileostomy. (D) Resected specimen.

A 70-year-old man presented to the emergency department with acute abdominal pain. He had no prior surgical history and no significant past medical history. Contrast-enhanced computed tomography revealed a whirl sign accompanied by marked dilatation of the small intestine (Fig. 1). Based on these findings, a diagnosis of small bowel volvulus was made, and emergency surgery was performed. Extensive small-bowel resection was required, and jejunostomy and ileostomy were created intraoperatively. Both the jejunostomy and ileostomy were constructed as end stomas, resulting in a double-stoma configuration (Fig. 2).

Postoperatively, the patient experienced extremely high jejunostomy output, requiring frequent ostomy bag changes. Due to the difficulty with stoma management and fluid control, the jejunostomy was closed 4 months after the initial surgery. At the time of stoma closure, the residual bowel length was markedly limited. Therefore, to maximize the effective use of the remaining intestine, an end-to-end anastomosis between the jejunal and ileal stumps was performed using the Albert–Lembert suture technique. The length of the remaining small intestine was approximately 20 cm from the ligament of Treitz to the terminal ileum. The entire colon was preserved and remained in continuity. Home parenteral nutrition was initiated to maintain the patient's nutritional and hydration status. The patient was managed with home parenteral nutrition and was not treated in a specialized IF unit. Despite treatment with antidiarrheal agents, he continued to experience severe diarrhea, resulting in dehydration and poor symptom control.

At 1 year and 4 months after the initial surgery, the patient developed acute cholecystitis due to gallstones and subsequently underwent laparoscopic cholecystectomy. During a 4-year postoperative period, the patient experienced recurrent CRBSI, with a total of 18 episodes. Moreover, frequent nocturnal bowel movements caused significant sleep disturbance despite ongoing antidiarrheal therapy. Prior to initiation of teduglutide, the patient developed selenium deficiency and required regular supplementation.

**Figure 3.**
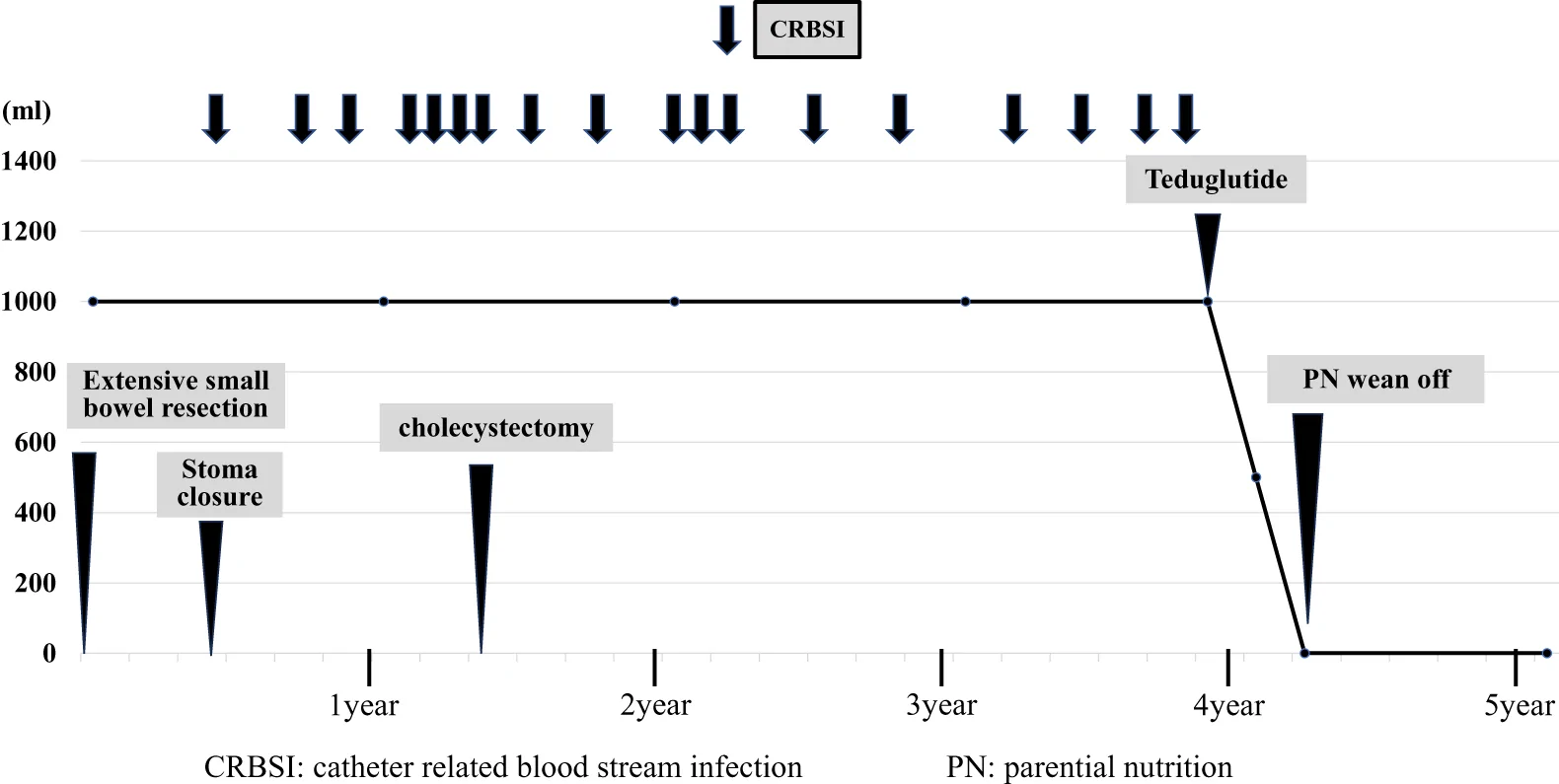
Clinical course and overview of the amount of parenteral support.

Considering the refractory nature of his condition and repeated complications related to long-term PN, teduglutide therapy was initiated 4 years after the initial surgery. Teduglutide was administered subcutaneously at a dose of 0.05 mg/kg/day, in accordance with standard clinical practice. Shortly after initiation of teduglutide, there was gradual clinical improvement. Initially, the patient required 1000 mL/day of PN. After starting teduglutide, the patient’s intestinal absorptive function gradually improved, allowing a stepwise reduction in parenteral support. Specifically, the PN volume was reduced to 500 mL/day at 2 months after treatment initiation. At 4 months, PN was completely discontinued, as the patient’s laboratory findings, body weight, and oral intake had stabilized. By 4 months of treatment, the stool consistency improved from watery to semi-formed, and the frequency of bowel movements decreased markedly from approximately 10 to 3–5 times per day. This reduction in bowel frequency led to improved nocturnal sleep quality. In parallel, teduglutide therapy reversed progressive weight loss and contributed to the stabilization and maintenance of body weight. Visceral protein markers also improved during this period (Fig. 3). At present, teduglutide therapy has been continued for 4 years. Although occasional supplemental intravenous fluid administration has been required, the patient’s condition has remained stable throughout the follow-up period.

After weaning from PN, no further CRBSI occurred, and no adverse events related to teduglutide were detected. The patient’s general condition continued to improve, and daily subcutaneous teduglutide therapy was maintained. At 6 months after the initiation of teduglutide, upper gastrointestinal endoscopy and colonoscopy were performed for surveillance, and they revealed no evidence of polyps or neoplastic lesions. Histological examination of biopsy specimens obtained from the remnant small intestine demonstrated elongated intestinal villi, consistent with improved mucosal adaptation (Supplemental Digital Content Material 1, available at: https://links.lww.com/IJSCR/A115).

We evaluated the QoL using the disease-specific SBS-QoL questionnaire, a validated instrument comprising 17 items with a total score ranging from 0 to 170; higher scores indicate poorer QoL. Before initiating teduglutide therapy, the patient showed a markedly impaired QoL, particularly in domains related to gastrointestinal symptoms, sleep disturbance, fatigue, and daily activities. One 1 year after initiating teduglutide, there was substantial improvement across multiple SBS-QoL domains (Supplemental Digital Content Material 2, available at: https://links.lww.com/IJSCR/A116).

## Discussion

SBS-IF is a debilitating condition associated with lifelong dependence on PN, recurrent CRBSI, and marked impairment of QoL. In this report, we describe the case of a patient with an extremely short residual small-bowel length (approximately 20 cm) secondary to small-bowel volvulus who achieved complete independence from PN following teduglutide treatment. This favorable clinical course was accompanied by reductions in stool frequency, stabilization of body weight, the elimination of CRBSI, and a significant improvement in disease-specific QoL.

The efficacy of teduglutide has been demonstrated in pivotal randomized controlled trials, including the STEPS and STEPS-2 studies, which reported clinically significant reductions in PN and intravenous fluid requirements, with a subset of patients achieving enteral autonomy^[^8,10^]^. Subsequent Phase III studies conducted in Japan confirmed that the pharmacokinetics, efficacy, and safety of teduglutide in Japanese patients were comparable to those in Western populations, thereby supporting its use in routine clinical practice^[^11^]^.

In Japan, postmarketing and real-world evidence concerning teduglutide has gradually accumulated. For instance, a nationwide, all-case postmarketing surveillance conducted across 102 sites and led by Dr. Wada and colleagues demonstrated that a substantial proportion of patients achieved clinically significant reductions in parenteral support requirements, with 42.4% achieving ≥20% reduction and 11.9% successfully discontinuing parenteral support altogether in this 6-month interim analysis while maintaining an acceptable safety profile^[^12^]^.

Nevertheless, despite these encouraging results, there is limited clinical experience with teduglutide in patients with ultrashort bowel – generally defined as a residual small bowel length of <30 cm – in Japan. Most reported Japanese cases involve patients with relatively longer remnant bowel or Crohn’s disease–related SBS, and data specifically addressing ultrashort bowel anatomy are scarce. In this context, evidence from Western real-world cohorts provides important complementary insights.

A single-center experience from the United States, reported by Lam *et al*^[^13^]^ described the outcomes of teduglutide therapy in adult patients with SBS-IF, including individuals with extremely short residual small bowel lengths. In that cohort, approximately 60% of patients achieved complete independence from PN and/or intravenous fluids, and the PN volume was reduced in almost all treated patients. Importantly, successful weaning from parenteral support was observed even in patients with very short residual bowel, provided that the colon was preserved. These data suggest that teduglutide exerts clinically significant effects even in ultra-SBS, thereby supporting the biological plausibility of the favorable outcome observed in the present case.

An important strength of the present case is the comprehensive evaluation of patient-centered outcomes. Although early clinical trials primarily focused on reductions in PN volume as surrogate endpoints, disease-specific QoL has emerged as a crucial therapeutic target in SBS-IF. Accordingly, using the validated SBS-QoL questionnaire^[^2^]^, we observed substantial improvements across multiple domains, including gastrointestinal symptoms, sleep disturbance, fatigue, and daily activities. These improvements closely paralleled reductions in stool frequency and infusion burden, highlighting the clinical relevance of QoL evaluation in this population.

Furthermore, stabilization and maintenance of body weight represent clinically relevant outcomes in patients with SBS-IF. Progressive weight loss indicates insufficient absorptive capacity and ongoing catabolic stress, and is associated with frailty and poor prognosis, particularly in elderly patients. In the present case, teduglutide treatment not only halted progressive weight loss but also enabled sustained body weight maintenance after the discontinuation of PN. This finding suggests that improved intestinal adaptation was sufficient to meet caloric and fluid requirements through enteral intake alone, thus positioning body weight maintenance as a vital marker of functional intestinal recovery beyond PN reduction alone.

From a mechanistic viewpoint, the benefits observed in this case are consistent with the known intestinotrophic effects of GLP-2, including increased villus height, improved mucosal absorptive capacity and intestinal blood flow, and strengthened epithelial barrier function. Histological findings of elongated intestinal villi support these mechanisms. Emerging evidence also suggests potential effects of GLP-2 signaling on intestinal immune function and gut microbiota composition, which may contribute to a reduced occurrence of infectious complications, although direct human data remain limited^[^14,15^]^.

Several limitations of this study should be acknowledged. First, this is a single-case report, and its findings may not be generalizable to all patients with SBS-IF. Second, the entire colon was preserved and remained in continuity, a factor known to improve intestinal adaptation and responsiveness to teduglutide. Therefore, the favorable outcome observed in this case may not be directly applicable to patients without colon continuity. Finally, long-term follow-up is necessary to confirm the durability of PN independence and to monitor for potential adverse events, including intestinal neoplasia.

Nevertheless, despite these limitations, this case provides valuable evidence that teduglutide may be effective even in patients with an ultrashort residual small-bowel length. In regions such as Japan, where experience with teduglutide in ultra-SBS is limited, this report adds clinically relevant insights and supports consideration of teduglutide as a therapeutic option aimed not only at reducing parenteral support but also at improving QoL and achieving significant functional recovery.

## Data Availability

Not applicable.
